# Morphological Aspects and Immunophenotypic Profiles of Mammary Carcinomas in Benign-Mixed Tumors of Female Dogs

**DOI:** 10.1155/2012/432763

**Published:** 2012-09-13

**Authors:** Gustavo Meirelles Ribeiro, Angélica Cavalheiro Bertagnolli, Rafael Malagoli Rocha, Geovanni Dantas Cassali

**Affiliations:** ^1^Department of Medical Sciences, Federal University of Ouro Preto, Ouro Preto, MG, Brazil; ^2^Faculty of Health Sciences, University of Vale do Rio Doce, Governador Valadares, MG, Brazil; ^3^Laboratory of Investigative Pathology, Department of Anatomic Pathology, Hospital A.C. Camargo, São Paulo, SP, Brazil; ^4^Laboratory of Comparative Pathology, Department of General Pathology, ICB-UFMG, 31270-901 Belo Horizonte, MG, Brazil

## Abstract

Carcinoma in benign-mixed tumor (CBMT) is common in the female canine mammary gland and comprises malignant epithelial between benign mesenchymal elements. This study investigated the morphological aspects of 29 CBMT and their immunophenotypical profiles, by using an immunohistochemistry panel based on five molecular markers—estrogen receptor (ER), progesterone receptor (PR), human epidermal growth factor receptor 2 (HER2), cytokeratin 5 (CK5), and human epidermal growth factor receptor 1 (EGFR). From these, CBMT was classified into four subtypes: luminal A, luminal B, HER2-like, basal-like, and normal. “*In situ*” and invasive carcinomatous components were analyzed and compared. Histological grade I carcinoma was observed in 16 cases (55.2%) of the tumors analyzed, grade II in 10 cases (34.5%), and grade III in three cases (10.3%). The invasive carcinomatous component has shown, more frequently, luminal A (12/29 cases, 41.4%), followed by basal-like phenotype (8/29 cases, 27.6%). There was high concordance between immunophenotypical profiles of the *in situ* and invasive carcinomatous components (kappa coefficient = 0.816, *P* < 0.001). We concluded that CBMT predominantly has features of low-grade neoplasms of malignancy. The various immunophenotypic profiles suggest the origin of these lesions in more than one cell type (luminal and myoepithelial).

## 1. Introduction

Mammary glands are the most frequent site of neoplasias in female dogs, and mammary cancer is one of the leading causes of death in these animals [1, 2]. These tumors have similarities to human breast neoplasms, and therefore, their etiopathogeny and biological features are of great interest [3, 4].

Mixed tumors (MTs) are histologically characterized by a mixture of epithelial components (ductal and/or acinous cells and myoepithelial cells) within an apparent mesenchymal stroma capable of producing various amounts of myxoid, chondroid, and bone tissues. These neoplasias can be benign-mixed tumors or can undergo malignant transformation, giving rise to carcinomas in benign-mixed tumors (CBMTs) [5, 6].

Several studies have investigatedthe relationship betweenprognostic factors and breast carcinomasin female dogs,but little is knownabout the biological behaviorand prognosisof these neoplasms [7, 8]. 

The molecular-based classification system adopted for breast cancer is a valuable tool for assessing prognosis and investigating similarities between the canine and human tumor types. According to thisdata,at least five different molecular subtypes ofhuman breast carcinomas were identified, based on gene expression profiling:luminalA,luminalB,HER2,basal-like, andnormal type carcinoma, all of whichdiffer in theirpathological and clinical profiles [9, 10]. However, there are few studies for the molecular characteristics of breast tumors in female dogs [11, 12].

In the present study, the morphological and immunophenotypic features of invasive carcinoma components in CBMT of the female dogs were analyzed and compared with *in situ* carcinoma component counterparts. From these findings, we determined the immunophenotypic profiles of the studied tumours. 

## 2. Materials and Methods

### 2.1. Specimen Selection

Twenty nine (*n* = 29) tissue samples of CBMT were obtained from female dogs (several breeds) that underwent surgery at the Veterinary School Hospital of Universidade Federal de Minas Gerais (EV/UFMG), Belo Horizonte, Brazil. The procedures performed in this study were submitted and approved by the Ethics Committee in Animal Experimentation of this institution (CETEA/UFMG) under protocol no. 123/2009.

The morphological criteria used for the diagnosis of the lesions were proposed by Misdorp et al. [13]. Inclusion criteria were the integrity of paraffin blocks from each case and prior diagnosis of carcinoma in a benign-mixed tumor. Exclusion criteria included damage to paraffin blocks, diagnostic doubts, other diagnoses, and samples demonstrating fixation and processing artifacts. All samples were subjected to histological processing by routine techniques and were embedded in paraffin blocks. Six consecutive histological sections (4 *μ*m) were cut from the paraffin block of each case, previously considered as representative of the lesion. Hematoxylin and eosin staining (H&E) was performed on the first section, and then neoplasia classification was carried out according to the protocol of Misdorp et al. [13]. Subsequent sections were used for immunohistochemistry.

In the malignant epithelial component, the following parameters were observed: histological grade [15], mitotic index (Table 1), presence of tumoral necrosis, presence of *in situ* (malignant noninvasive) proliferation, and the presence of stromal and vascular invasion. Stromal invasion was diagnosed when the tumor reached or exceeded the stroma of the mixed tumor studied. The presence of neoplastic emboli within the spaces covered by endothelium was established to determine vascular invasion. The malignant epithelial component *in situ* was classified according to the Consensus Conference on the classification of ductal carcinoma *in situ *[16]. The lesions were graduated according to Lagios [17] in high, intermediate, and low degrees. These criteria are also consistent with recently published consensus in veterinary cancer [3]. 

In the stromal components, the following parameters were evaluated: presence of stromal elements (chondroid and myxoid matrix, cartilage, bone tissue, and bone marrow) and investigation of the morphological features for malignancy. Each case was examined by two pathologists, and discordant results were discussed with a third pathologist for consensus.

### 2.2. Immunohistochemistry

Consecutive 4 *μ*m sections were cut and mounted on silanized slides. Tissue sections were deparaffinized and rehydrated in graded ethanol. The ER, PR receptors, and CK5 antigens were retrieved using a citrate buffer (pH 6.0) and heated for four minutes in a Pascal pressure chamber (DAKO). The slides were immersed in 3% hydrogen peroxide for 30 minutes (three baths for 10 minutes) to ensure endogenous peroxidase blocking. The histological sections were incubated in a humid chamber for 18 hours with a primary antibody. Antigens were detected using the Novolink System Max Polymer (Leica) for 30 minutes at 37°C. 

HER2 and EGFR antigens were retrieved using solution retrieval (DAKO), at pH 6.0 for 20 minutes in a water bath at 98°C. The endogenous peroxidase was blocked by immersion in 3% hydrogen peroxide for 15 min. Samples were incubated in a humid chamber for 14 hours (overnight) with a primary antibody and amplified with the Advance HRP link (DAKO). 

For all slides, 3, 3′-diaminobenzidine (DAB) was used as a chromogen. Sections were counterstained with Mayer's hematoxylin, dehydrated, and mounted in a synthetic medium.  Table 2 lists the characteristics of the primary antibodies. Canine mammary tumor samples, known to express all markers, were used as positive controls. Negative controls were obtained by omitting the primary antibody.

Cells were considered positive for ER and PR markers when more than 5% of the cell nucleus was stained [18]. The HER2 marker followed the pattern established by the American Society of Clinical Oncology/American College of Pathology [19]. Cases were considered positive for EGFR when more than 10% of the cell membrane was stained [20]. CK5 expression was scored positive if any (weak or strong) cytoplasmic and/or membranous tumoral cell was observed [21].

Molecular profiles of the samples analyzed were defined as follows: luminal A (ER+ and/or PR+, HER2−, any EGFR, and CK5); luminal B (ER+ and/or PR+, HER2+, any EGFR, and CK5); HER2-like (ER− and PR−, HER2+, any EGFR, and CK5); basal-like (ER− and/or PR−, HER2−, EGFR, and/or CK5+); normal (negative for all markers) [22].

### 2.3. Statistical Analysis

Along with establishing the molecular profiles of the sample cases, a descriptive analysis of the absolute and relative frequencies of their morphological and phenotypical features was carried out.

Fisher's exact probability test (*P* < 0.05) was used to test possible associations between the studied features. Analysis of concordance between the molecular profile of *in situ* components and invasive components was carried out using the kappa statistic index and kappa significance test. The conventional 5% level of significance was used to define the statistical significance (*P* < 0.05). Analyses were performed using STATA 9.0 software (StataCorp LP).

## 3. Results

### 3.1. Morphology

CBMTs of the canine mammary gland were defined as carcinomatous cells occurring in a benign-mixed tumor [5, 6]. The diagnosis of carcinoma was predominantly based on the presence of a focus of atypical epithelial cells, often demonstrating infiltrative growth characterized by the presence of clusters of tumor cells penetrating the stroma. The presence of an *in situ* (malignant, noninvasive) component was evaluated. An *in situ* malignant epithelial component was identified in 15 of the 29 samples (51.7%). Nine of these were of the solid histological type (60%), three were solid/cribriform (20%), two were micropapillary (13.3%), and one sample was papillary/micropapillary (6.7%). The most frequent histological grades were for II (73.3%; 11 cases) and for I (20%; three cases).

The majority of invasive carcinomas were of low histological grade (16/29 samples; 55.2%) and low mitotic index (21/29 samples; 72.4%) as demonstrated in Tables 3 and 4, respectively. 

Focal and discrete necrosis was observed in seven cases (24.1%). With the exception of one case (3.4%), no vascular invasion and squamous metaplasia were noted in the samples. No malignant transformation was observed in the stromal component of any lesion. Myxoid matrix was evident in 10 cases (34.5%) and myxoid/chondroid matrix in 19 cases (65.5%). In the stromal component, only a mesenchymal matrix (myxoid or myxochondroid) was observed in 19 (65.6%) samples; both matrix and cartilage were observed in four (13.8%) samples; matrix, cartilage and bone in three (10.3%) samples; matrix, cartilage, and bone with bone marrow in three (10.3%) samples (Table 5).

No significant statistical association was demonstrated between profiles identified in the epithelial component and stromal pattern (myxoid and myxoid/chondroid) (*P* = 0.773), or between profiles of the epithelial component and stromal elements (*P* = 0.48).

### 3.2. Immunohistochemical Profile

Among the *in situ* malignant epithelial components, the luminal A profile accounted for 60% of the tumors (nine out of 15 cases), followed by the HER2-like profile (20%; three cases), the luminal B profile (13.3%; two cases), and the basal profile (6.7%; one sample), according to the criteria adopted for molecular profiling. 

**Figure 1:**
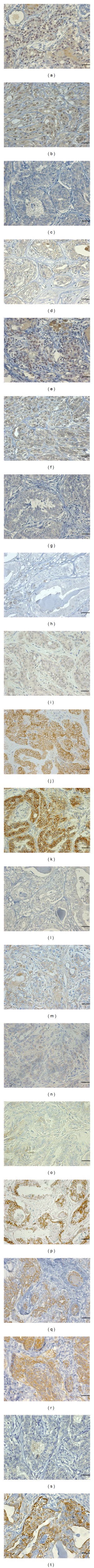
Immunophenotypic profiles of mammary carcinomas in benign-mixed tumors of female dogs' mammary gland: *Canis familiaris*. (a)–(d) ER staining; (e)–(h) PR staining; (i)–(l) HER2 staining; (m)–(p) EGFR staining; (q)–(t) CK5 staining. Each column represents a distinct molecular subtype. From left to right: luminal A (case 13—RE: positive, RP: positive, HER2: negative, EGFR: negative, and CK5: positive). Luminal B (case 21—RE: positive, RP: positive, HER2: positive, EGFR: negative, and CK5: positive), HER2 (case 20—RE: negative, RP: negative, HER2: positive, EGFR: negative, and CK5: negative), and basal-like (case 17—RE: negative, RP: negative, HER2: negative, EGFR: positive, and CK5: positive). Advance HRP visualization method streptavidin-biotin complex method. Mayer's hematoxylin counterstain Bar: 25 *μ*m.

In invasive carcinomas, the luminal A profile was predominant, accounting for 41.4% (12 out of 29 cases), followed by the basal-like profile (27.6%; eight cases), the HER 2 profile (17.2%; five cases), and the luminal B profile (13.8%; four samples) (Table 6). In terms of the invasive component, myoepithelial cell markers CK5 and/or EGFR were expressed in 27 cases. Fifteen samples (51.7% of all cases) were positive for both markers, 10 cases (3.4%) were positive for CK5 alone, and two (6.9%) were positive for EGFR alone. There were no statistically significant correlations between the immunophenotypical profiles of the invasive malignant epithelial component in mixed tumors and histological grade (*P* = 0.735) or mitotic index (*P* = 0.076).  Figure 1 shows some profiles studied.

In terms of invasive carcinoma, cases in which the *in situ* component was identified (*n* = 14), the luminal A immunophenotypical profile accounted for 50% of cases, the basal-like profile accounted for 35.8%, the luminal B accounted for 7.1%, and the HER2-like profile was evident in 7.1% of the samples. The kappa coefficient showed a high concordance between immunophenotypical profiles of the *in situ* malignant epithelial component and invasive malignant epithelial component (kappa coefficient = 0.816, *P* < 0.001) (Table 7).

## 4. Discussion

The morphological analysis of 29 cases of carcinoma in mixed tumor from female dogs revealed a predominantly low histologic grade and a low mitotic index. Invasive carcinoma was predominantly of histological grade I (55.2%). Our results are in agreement with those of Karayannopoulou et al. [23], which also showed a predominance of low-grade carcinoma in CBMT. Similar data was reported by Dutra et al. [14]. They showed that 75% of the cases were of histological grade I and that 80% of these had a five-year survival rate after prognosis. Other findings may indicate morphological characteristics of low aggressiveness of CBMT, including the absence of vascular invasion and little necrosis (focal and mild in 24.1% of cases). Further studies about CBMT revealed similar results [12, 24]. 

The rates of the immunophenotypic profile of the carcinomatous component (luminal A—41.4%; Basal-like—27.6%) are similar to those reported in other immunohistochemical studies in a variety of mammary tumors in female dogs (luminal A—44.8%, basal-like 29.2%) [11]. Sassi et al. demonstrated that the most frequent immunophenotypic profile was luminal B—48%, followed by basal-like (28%) [25]. These findings suggest that CBMT is a heterogeneous group of neoplasms with various immunoprofiles. The little differences between studies may be explained by the use of distinct immunohistochemical panels [25].

Sassi et al. [25] demonstrated the correlation between an immunophenotypic profile and the histological grade of mammary carcinomas in female dogs, but this was not evident in this study. One possible explanation for the differences in these results is the variety of histological types of carcinomas involved in this study.

Immunohistochemical analysis demonstrated the heterogeneity of profiles of tumors, whereby luminal A represented 41.1% and basal-like 27.6% of cases. The luminal A profile in humans has been described as a subtype related to a low histological grade and excellent prognosis [9]. 

CBMT of female dogs is comparable to the carcinoma ex-pleomorphic adenoma (CXPA) of the salivary gland of humans [7]. The malignant epithelial component of a carcinoma ex-pleomorphic adenoma of the salivary gland can be classified into two main groups: carcinoma with luminal differentiation (ductal epithelium), for example, adenocarcinoma not otherwise specified (NOS), and carcinoma with nonluminal (myoepithelial) differentiation, for example, adenoid cystic carcinoma [26]. Interestingly, the proportion of luminal tumors of salivary glands CXPA found by the authors (75.0%) was similar to the combination of the profiles (luminal A, luminal B, and HER2-like = 72.4%) identified in this study. The various immunophenotypic profiles observed in present study could reflect the different origin of the malignant cells (epithelial/myoepithelial).

An interesting point is the positivity for EGFR in luminal A (eight cases, 47% of the positive cases) and luminal B (two cases, 11.8% of the positive cases) immunoprofiles, since this is a marker present in benign myoepithelial cells. In a discussion about the expression of EGFR in the mammary tumors of dogs, Gamma et al. [27] proposed that the positivity for this marker would mean the immunophenotypic profile maintenance of original myoepithelial cells. A new interpretation for this phenomenon would be the upregulation of EGFR in epithelial cells (luminal) as a process of malignant tumor evolution [24].

Luminal A was the more frequent immunoprofile in *in situ* component of CBMT (60%). This finding is consistent with studies of *in situ* carcinoma in humans when the corresponding invasive carcinoma component is of low histological grade [28]. There was high agreement between the molecular profiles of *in situ* and invasive carcinoma components in CBMT.

The method of grading used in human carcinomas is reproducible, and similar models are found in veterinary studies [6, 29]. The recognition and diagnosis of mammary intraepithelial lesions in veterinary pathology has been a subject in the recent literature, either for the identification of these lesions adjacent to invasive carcinomas, or for the prospect of its use as experimental models about pathogenesis and prognosis of these lesions [30–32].

Studies using immunohistochemical panels for the molecular characterization of mammary carcinoma in female dogs are rare, and the results seem inconsistent when compared with the clinicopathological features of human carcinoma. Data is conflicting, as studies show better survival rates of patients with carcinomas of the profile of HER2-like and no correlation between the molecular profile and histological type [11, 25]. This conflicting data suggests that more complex studies with larger samples are needed to evaluate the same types of carcinomas, in order to indicate treatment strategies and to define experimental models for research.

## 5. Conclusions

We concluded that CBMT is predominantly of the luminal A immunophenotype and generally shows morphological characteristics that are associated with better prognosis in canine mammary neoplasms. The various immunophenotypic profiles suggest the origin of these lesions in more than one cell type (luminal and myoepithelial). In most cases, it is possible to identify intraepithelial nepolasia adjacent to invasive carcinomas. Also, the *in situ* carcinomas studied have similar immunophenotypic profile to their invasive counterparts.

## Figures and Tables

**Table 1:** Correlation between the mitosis number and mitotic index in carcinomas.

Number of mitosis/10 fields, magnification 40x (*)	Score	Classification
0–7	1	Low
8–16	2	Moderate
≥17	3	High

*Field diameter: 0.55 mm; field area: 0.26 mm^2^. Dutra et al., 2008 [14].

**Table 2:** Immunohistochemical panel of antibodies.

Antibody	Manufacturer	Clone	Concentration	Signal
ER	Dako	1D5	1 : 100	Nucleus
PR	Novocastra	IA6	1 : 20	Nucleus
HER2	Dako	A0485	1 : 180	Membrane
EGFR	Zymed	31G7	1 : 100	Membrane/cytoplasmic
CK 5	Novocastra	XM26	1 : 300	Cytoplasmic

**Table 3:** Histological grade of the invasive epithelial component (*n* = 29).

Grade	Frequency	%
I	16	55.2
II	10	34.5
III	3	10.3

Total	29	100

**Table 4:** Mitotic index of the invasive epithelial component (*n* = 29).

Mitotic index	Frequency	%
Low	21	72.4
Moderate	6	20.7
High	2	6.9

Total	29	100

**Table 5:** Frequency of the tissue types observed in the stromal component of carcinoma in mixed tumor (*n* = 29).

Mesenchymal component	Frequency	%
Myxoid matrix	19	65.6
Myxoid matrix and cartilage	4	13.8
Myxoid matrix, cartilage, and bone	3	10.3
Myxoid matrix, cartilage, and bone with bone marrow	3	10.3

Total	29	100

**Table 6:** Immunophenotypical profile distribution of the invasive component (*n* = 29).

Immunophenotypical profile	Frequency	%
Luminal A	12	41.4
Basal	8	27.6
HER2	5	17.2
Luminal B	4	13.8

Total	29	100

**Table 7:** Concordance between immunophenotypical profiles of *in situ* malignant epithelial component and invasive malignant epithelial component (*n* = 15).

Profile (*in situ*)	Profile (invasive)				Kappa coefficient (*P* value)
	Luminal A	Luminal B	HER2	Basal
Luminal A	5	0	0	1	0.816 (<0.001)
Luminal B	0	2	0	0	
HER2	0	1	4	0	
Basal	0	0	0	2	

## References

[1] Dobson JM, Samuel S, Milstein H, Rogers K, Wood JLN (2002). Canine neoplasia in the UK: estimates of incidence rates from a population of insured dogs. *Journal of Small Animal Practice*.

[2] Bravo D, Casallas PEC, Amaya JO (2010). Prevalencia de neoplasias en caninos en la universidad de los Llanos, durante 2004 a 2007. *Revista MVZ Córdoba*.

[3] Cassali GD (2000). *Aspectos morfológicos, imunohistoquímicos e citométricos de tumores mamários da cadela—aspectos comparativos com neoplasias da mama humana [Ph.D. thesis]*.

[4] Richards HG, McNeil PE, Thompson H, Reid SWJ (2001). An epidemiological analysis of a canine-biopsies database compiled by a diagnostic histopathology service. *Preventive Veterinary Medicine*.

[5] Moulton JE, Taylor DO, Dorn CR, Andersen AC (1970). Canine mammary tumors. *Pathologia veterinaria*.

[6] Cassali GD, Lavalle GE, De Nardi AB, Ferreira E, Bertagnolli AC, Estrela-Lima A (2011). Consensus for the diagnosis, prognosis and treatment of canine mammary tumors. *Brazilian Journal of Veterinary Pathology*.

[7] Genelhu MCLS, Cardoso SV, Gobbi H, Cassali GD (2007). A comparative study between mixed-type tumours from human salivary and canine mammary glands. *BMC Cancer*.

[8] Rungsipipat A, Tateyama S, Yamaguchi R, Uchida K, Miyoshi N, Hayashi T (1999). Immunohistochemical Analysis of c-yes and c-erbB-2 Oncogene Products and p53 Tumor Suppressor Protein in Canine Mammary Tumors. *Journal of Veterinary Medical Science*.

[9] Sørlie T, Perou CM, Tibshirani R (2001). Gene expression patterns of breast carcinomas distinguish tumor subclasses with clinical implications. *Proceedings of the National Academy of Sciences of the United States of America*.

[10] Sørlie T, Tibshirani R, Parker J (2003). Repeated observation of breast tumor subtypes in independent gene expression data sets. *Proceedings of the National Academy of Sciences of the United States of America*.

[11] Gama A, Alves A, Schmitt F (2008). Identification of molecular phenotypes in canine mammary carcinomas with clinical implications: application of the human classification. *Virchows Archiv*.

[12] Kurilj AG, Hohsteter M, Artukovié B, Severin K, Sostarié-Zuckermann IC, Beck A (2011). Histopathological evaluation and immunohistochemical study of estrogen receptor *α*, HER-2 and Ki-67 in canine neoplastic mammary lesions. *Veterinarski Arhiv*.

[13] Misdorp W, Else W, Hellmen E, Lipscomb TP (1999). Histological classification of the mammary tumors of the dog and the cat. *World Health Organization International Histological Classification of Tumors of Domestic Animals*.

[14] Dutra AP, Júnior GMA, Schmitt FC, Cassali GD (2008). Assessment of cell proliferation and prognostic factors in canine mammary gland tumors. *Arquivo Brasileiro de Medicina Veterinaria e Zootecnia*.

[15] Elston CW, Ellis IO (1991). Pathological prognostic factors in breast cancer. I. The value of histological grade in breast cancer: experience from a large study with long-term follow-up. *Histopathology*.

[16] (1997). Consensus conference on the classification of ductal carcinoma in situ. *Human Pathology*.

[17] Lagios MD (1990). Duct carcinoma in situ: pathology and treatment. *Surgical Clinics of North America*.

[18] Millanta F, Calandrella M, Bari G, Niccolini M, Vannozzi I, Poli A (2005). Comparison of steroid receptor expression in normal, dysplastic, and neoplastic canine and feline mammary tissues. *Research in Veterinary Science*.

[19] Wolff AC, Hammond MEH, Schwartz JN (2007). American society of clinical oncology/college of American pathologists guideline recommendations for human epidermal growth factor receptor 2 testing in breast cancer. *Archives of Pathology and Laboratory Medicine*.

[20] Rakha EA, El-Sayed ME, Green AR, Lee AHS, Robertson JF, Ellis IO (2007). Prognostic markers in triple-negative breast cancer. *Cancer*.

[21] Nielsen TO, Hsu FD, Jensen K (2004). Immunohistochemical and clinical characterization of the basal-like subtype of invasive breast carcinoma. *Clinical Cancer Research*.

[22] Carey LA, Perou CM, Livasy CA (2006). Race, breast cancer subtypes, and survival in the Carolina Breast Cancer study. *Journal of the American Medical Association*.

[23] Karayannopoulou M, Kaldrymidou E, Constantinidis TC, Dessiris A (2005). Histological grading and prognosis in dogs with mammary carcinomas: application of a human grading method. *Journal of Comparative Pathology*.

[24] Bertagnolli AC, Ferreira E, Dias EJ, Cassali GD (2011). Canine mammary mixed tumours: immunohistochemical expressions of EGFR and HER-2. *Australian Veterinary Journal*.

[25] Sassi F, Benazzi C, Castellani G, Sarli G (2010). Molecular-based tumour subtypes of canine mammary carcinomas assessed by immunohistochemistry. *BMC Veterinary Research*.

[26] Altemani A, Martins MT, Freitas L, Soares F, Araújo NS, Araújo VC (2005). Carcinoma ex pleomorphic adenoma (CXPA): immunoprofile of the cells involved in carcinomatous progression. *Histopathology*.

[27] Gama A, Gärtner F, Alves A, Schmitt F (2009). Immunohistochemical expression of epidermal growth factor receptor (EGFR) in canine mammary tissues. *Research in Veterinary Science*.

[28] Tamimi RM, Baer HJ, Marotti J (2008). Comparison of molecular phenotypes of ductal carcinoma in situ and invasive breast cancer. *Breast Cancer Research*.

[29] Goldschmidt MH, Peña L, Rasotto R, Zappulli V (2011). Classification and grading of canine mammary tumors. *Veterinary Pathology*.

[30] Antuofermo E, Miller MA, Pirino S, Xie J, Badve S, Mohammed SI (2007). Spontaneous mammary intraepithelial lesions in dogs—a model of breast cancer. *Cancer Epidemiology Biomarkers and Prevention*.

[31] Mouser P, Miller MA, Antuofermo E, Badve SS, Mohammed SI (2010). Prevalence and classification of spontaneous mammary intraepithelial lesions in dogs without clinical mammary disease. *Veterinary Pathology*.

[32] Ferreira E, Gobbi H, Saraiva BS, Cassali GD (2011). Histological and immunohistochemical identification of atypical ductal mammary hyperplasia as a preneoplastic marker in dogs. *Veterinary Pathology*.

